# On‐Surface Synthesis of Porous Carbon Nanoribbons on Silver: Reaction Kinetics and the Influence of the Surface Structure

**DOI:** 10.1002/cphc.201900347

**Published:** 2019-09-03

**Authors:** Maximilian Ammon, Martin Haller, Shadi Sorayya, Sabine Maier

**Affiliations:** ^1^ Department of Physics Friedrich-Alexander-Universität Erlangen-Nürnberg Erwin-Rommel-Straße 1 91058 Erlangen Germany

**Keywords:** molecular self-assembly, on-surface reactions, scanning probe microscopy, surface chemistry, Ullmann-type reaction

## Abstract

We report on the influence of the surface structure and the reaction kinetics in the bottom‐up fabrication of porous nanoribbons on silver surfaces using low‐temperature scanning tunneling microscopy. The porous carbon nanoribbons are fabricated by the polymerization of 1,3,5‐tris(3‐bromophenyl)benzene directly on the Ag surface using an Ullmann‐type reaction in combination with dehydrogenative coupling reactions. We demonstrate the successful on‐surface synthesis of porous nanoribbons on Ag(111) and Ag(100) even though the self‐assemblies of the intermediate organometallic structures and covalently‐linked polymer chains are different on both surfaces. Furthermore, we present the formation of isolated porous nanoribbons by kinetic control. Our results give valuable insights into the role of substrate‐induced templating effects and the reaction kinetics in the on‐surface synthesis of conformationally flexible molecules.

## 1 Introduction

On‐surface reactions of polycyclic hydrocarbons on metallic substrates have been widely employed to fabricate atomically precise carbon nanostructures, e. g. graphene nanoribbons (GNR) and porous graphene, in a bottom‐up fashion.^1^ Thereby, the molecular self‐assemblies of the precursor molecules and intermediates in on‐surface reactions depend strongly on the molecule‐substrate interactions, which vary with the surface reactivity, adsorption energy, the crystal lattice, and potential surface reconstructions. Hence, often subtle differences can lead to significant altered self‐assemblies and reaction products of the same molecule on different surfaces. An illustrative example is the on‐surface synthesis of graphene nanoribbons from dibromobianthracene derivatives (10,10’‐dibromo‐9,9’‐bianthryl). While the synthesis of armchair graphene nanoribbons (7‐AGNR) from these precursors has been shown to work on substrates such as Au(111),^2^ Au(110),^3^ or Ag(111),^2^, ^4^ it surprisingly results in chiral (3,1)‐GNRs on Cu(111)^5^ and nanographenes on Cu(110),^6^ however.

**Scheme 1:**
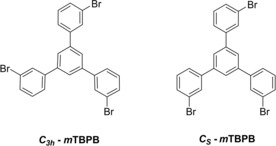
1,3,5‐tris(3‐bromophenyl)benzene (*m*TBPB) conformers with *C_3h_*‐and *C_s_*‐symmetry, respectively.

**Scheme 2:**
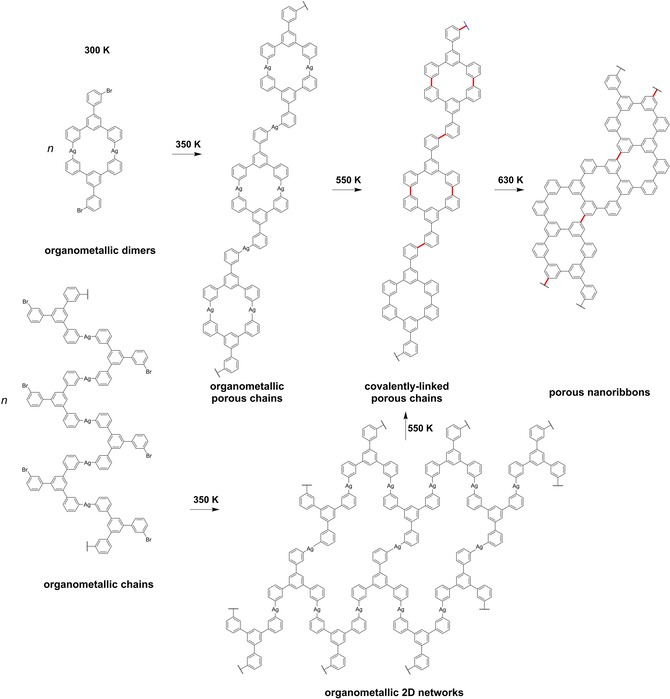
Reaction pathway of *m*TBPB on Ag(100) showing the most frequently observed structures. The reaction of *m*TBPB from the organometallic porous chains to the porous nanoribbons is similar on Ag(100) and Ag(111). On Ag(111), organometallic cyclic aggregates and distorted loops are additionally observed instead of organometallic 2D networks (see Figure 3a).^7^

In this study, we report on the critical role of the surface structure and the reaction kinetics in the bottom‐up fabrication of porous nanoribbons on Ag. We compare the reaction pathway of 1,3,5‐tris(3‐bromophenyl)benzene (*m*TBPB, Scheme 1) on Ag(111) and Ag(100) based on high‐resolution scanning tunneling microscopy (STM) experiments at low temperatures. The *m*TBPB molecule consists of three *m*‐phenylene rings. The conformation of the *m*TBPB can be switched between a *C_3h_*‐ and *C_s_*‐symmetry by a rotation of the *m*‐phenylene rings along the σ‐bonds to the central phenyl ring on Ag(111),^7^ which gives rise to a conformational flexibility of adsorbed *m*TBPB. In a first reaction step, polymer chains with periodic pores form from *C_s_*‐conformers after an Ullmann‐type coupling reaction. Thereby, the preferred linear intermolecular C−Ag−C bonds in the intermediate organometallic complexes after debromination template the high conformational selectivity of *C_s_‐*conformers in the polymer chains. In a second reaction step, the conformational flexibility of *m*TBPB facilitates the formation of porous carbon nanoribbons from the polymer chains with periodic pores on Ag(111)^7^ and Ag(100). The zigzag‐shaped ribbons are constructed *via* the flipping of *m*‐phenylene units in combination with C−C bond formations through dehydrogenation reactions, see Scheme 2.

The motivation to understand the nanoribbon formation on the Ag(111) vs. Ag(100) lattice results from the observation that organometallic intermediates in on‐surface synthesis are in general sensitive to the surface structure, as the surface registry of the adatoms can direct their alignment.^8^ Ag(100) possesses a symmetry‐mismatched four‐fold structure with respect to the *C_3h_‐*conformers, as opposed to the three‐fold symmetry of the Ag(111). Correspondingly, differences in the organometallic intermediates are expected on the two Ag lattices, if the lattice registry of Ag adatoms drives the structure oft he organometallic overlayers. At present, a majority of on‐surface synthesis reactions on Ag are reported on the (111) facet, although Ullmann‐type coupling also successfully proceeds on Ag(110)8b, 9 and Ag(100)^10^ surfaces. Therefore, this study will provide further interesting insights into Ullmann‐type reactions on Ag(100), which has rarely been addressed although the Ullmann‐type reaction is a workhorse in on‐surface synthesis.

## 2 Results and Discussion

### 2.1 Influence of the Surface Structure: Intermediate Organometallic Structures

In the following, we compare the reaction pathway of *m*TBPB towards porous nanoribbons at sub‐monolayer coverage on Ag(111) vs. Ag(100). The reaction pathway of *m*TBPB towards porous nanoribbons on Ag(111) is in detail discussed in Ref. [7]. Hence, we focus here in particular on Ag(100).

**Figure 1:**
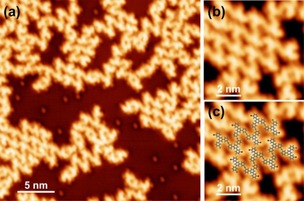
Organometallic structures of partially debrominated *m*TBPB adsorbed at room temperature on Ag(100): (a) Overview STM image, (b) zoom on self‐assembled organometallic zig‐zag chains with a tentative structural model in (c). STM parameters: (a) −50 mV, 150 pA; (b–c) −50 mV, 250 pA.

Upon adsorption onto the Ag(100) surface at sub‐monolayer coverage at room temperature (RT, 300 K), *m*TBPB is partially debrominated. The bright protrusions on the Ag terraces and at the periphery of the *m*TBPB structures correspond to cleaved Br atoms, as seen in the STM image in Figure 1a. Mostly organometallic dimers and zig‐zag chains built from *C_s_*‐conformers were observed. Along the zig‐zag chains, the molecules bind *via* linear intermolecular C−Ag−C bonds. The chains self‐assemble in small islands *via* H−Br hydrogen bonding and Br−Br halogen bonding,^11^ as seen in the high‐resolution STM images in Figure 1b–c. The tentative model of the zig‐zag chains is overlaid in Figure 1c to guide the eye. A majority of *m*TBPB molecules adopt a *C_s_*‐symmetry, similar to the intermediate organometallic *m*TBPB structures previously reported on Ag(111).^7^ Hence, the Ag(100) surface also promotes a conformational selectivity towards the *C_s_*‐conformer.

**Figure 2:**
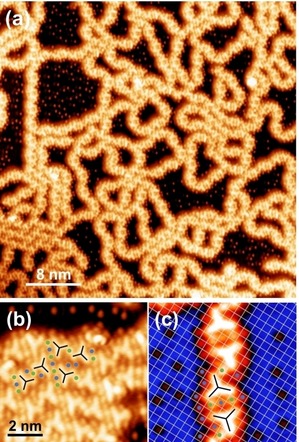
Organometallic structures of debrominated *m*TBPB on Ag(100) after annealing to 350 K: (a) Overview STM image, (b) zoom on an organometallic 2D network, and (c) zoom on a dimer chain. In (b–c) black tripods highlight the position of the debrominated *m*TBPB scaffold, blue dots and green dots the position of the Br and Ag metal centers, respectively. The STM images are recorded with a functionalized tip (likely Br‐terminated). STM parameters: (a–c) −50 mV, 50 pA.

**Figure 3:**
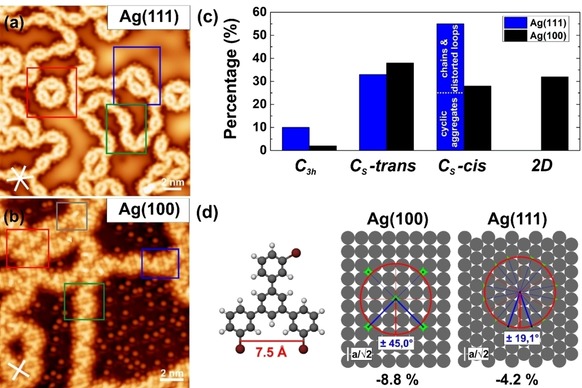
Comparison of the various organometallic *m*TBPB structures on Ag(100) vs. Ag(111): (a) Typical STM image of organometallic *m*TBPB‐structures on Ag(111), which shows chain segments (green frame), cyclic aggregates (red frame), and distorted loops (blue frame). (b) Typical STM image of organometallic *m*TBPB‐structures on Ag(100) composed of *C_3h_* conformers (gray frame), *C_S_‐trans* dimers (green frame), *C_S_‐cis* dimers (blue frame) and 2D networks (red frame). (c) Histogram showing the distribution of the various organometallic *m*TBPB‐structures. The percentages refer to the number of *m*TBPB precursor molecules. The evaluation includes 1355 monomers for Ag(111) and 1312 monomers for Ag(100) that are sampled from several positions on the surface. (d) Geometrical model explaining the alignment of the organometallic structures towards the substrate. Left: Structural model of the *m*TBPB calculated by DFT (B3LYP 6‐ 31G*); Right: Geometrical model of Ag(100) and Ag(111), respectively. The red circle represents the optimal Ag−Ag distance assuming a planar adsorption of the *m*TBPB (r=7.5 Å). Hence the hollow sites highlighted in green show preferred adsorption sites for the metal adatoms. This results in an alignment of neighboring Ag adatoms of ±45° (blue lines) with respect to the high‐symmetry axes on Ag(100) (red lines) and ±19,1° (blue lines) with respect to the high‐symmetry axes on Ag(111) (red lines). The experimentally observed alignments in the STM images fit perfectively with the suggested structural model. This corresponds to a misfit of a planar *m*TBPB of −8.8 % for Ag(100) and −4.2 % for Ag(111). The high‐symmetry directions of the Ag surfaces in the STM images are marked by the white lines in the left lower corner. STM parameters: (a) 50 mV, 50 pA; (b) −50 mV, 50 pA.

After annealing to 350 K, *m*TBPB is fully debrominated and we observe small patches of organometallic 2D assemblies as well as straight, curved, or branched organometallic chains, see Figure 2a. The formation of these structures indicates the availability of a sufficient amount of Ag adatoms. The organometallic 2D self‐assemblies are composed of zig‐zag chains of *C_s_*‐conformers (Figure 2b), while the chains are interlinked organometallic *C_s_‐m*TBPB dimers that have nanopores (Figure 2c). In contrast, only *C_s_‐m*TBPB organometallic structures based on dimers were observed on Ag(111) ranging from cyclic aggregates, deformed loops, and straight or branched chains, as seen by STM (Figure 3a)^7^ and Monte Carlo simulations.^12^ Thereby, the *C_s_‐m*TBPB dimers act as a template for the pores of the covalent cyclohexa‐*m*‐phenylene (CHP) rings in the polymer chains. In contrast, the 2D networks on Ag(100) do not provide a direct structural template for such pores and the porous nanoribbons.

We performed a statistical analysis of the various organometallic structures on Ag(100) and compared it to Ag(111), see Figure 3. Despite the structural diversity of the organometallic structures, we observe on both surfaces a high selectivity of more than 90 % towards *C_s_*‐conformers that is likely templated by the preferred linear intermolecular C−Ag−C bonds in these organometallic structures. On Ag(100), we observe both *C_s_‐cis* and *C_s_‐trans* dimers as well as 2D self‐assemblies in equal amounts. On Ag(111), the *C_s_‐cis* dimers dominate over the *C_s_‐trans* dimers, because *C_s_‐cis* dimers are included in cyclic aggregates and chains while *C_s_‐trans* dimers only occur in the chains.^7^ The different *m*TBPB conformers can be unambiguously identified in high‐resolution STM images recorded with functionalized tips (likely Br). Br‐terminated tips enhance the STM contrast and make the Br atoms and metal centers more visible, see Figure S1.

The cleaved Br atoms that adsorb on the Ag terraces can be used to determine the detailed adsorption geometry of the organometallic dimer chains on Ag(100). Br is chemisorbed on Ag(100) and preferentially adsorbs in fourfold hollow sites at sub‐monolayer coverage.^13^ The overlaid atomic lattice in Figure 2c (white grid, the points of intersection denote top sites) confirms that the bromines adsorb on the same type of adsorption sites. The orientation of the dimers with respect to the surface lattice is such that the Ag metal centers in a dimer and the surrounding Br at the periphery of the *m*TBPB can adsorb on the preferred fourfold hollow sites. Instead, the metal centers that connect the dimers seem to adsorb on bridge sites. The overview STM image in Figure 2a confirms that most of the organometallic dimers having pores appear in two orientations at approximately right angles, which supports that their alignment is directed by the high‐symmetry axes of the Ag(100) surface lattice. Indeed, an approximated distance of neighboring metal adatoms of 7.5 Å determined from a planar molecular geometry yields the best fit to the (100) surface lattice if the orientation is 45° (blue lines) with respect to the high‐symmetry axes (red lines), see Figure 3d. Similarly, the smallest misfit of neighboring metal adatoms is expected for a rotation of ±19,1° with respect to the high‐symmetry axes on the Ag(111), which is in good agreement with the experimentally observed ±22°.^7^ This results in an adatom‐separation of √(7/2) a=7.65 Å with a=4.09 Å on Ag(111). In conclusion, the organometallic structures on Ag(111) and Ag(100) are found to be driven by the registry of Ag adatoms with the surface atoms and hence different organometallic structures are observed on the two lattices. The strong templating effect of the metal centers explains, why on Ag(100), in contrast to Ag(111), no cyclic aggregates composed of three *C_s_‐trans* dimers are observed.

### 2.2 Influence of the Surface Structure: Covalently‐linked Porous Chains and Nanoribbons

**Figure 4:**
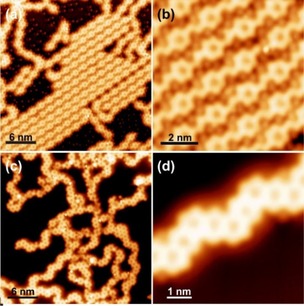
(a–b) Covalently‐linked *m*TBPB chains with periodic pores on Ag(100) after annealing at 550 K: (a) overview image and (b) zoom on a self‐assembled island formed from polymer chains of left‐ and right‐hand chirality (c–d) Overview and detailed image of porous nanoribbons after annealing to 630 K on Ag(100). STM parameters: (a, c) −50 mV, 150 pA; (b) −50 mV, 400 pA; (d) −50 mV, 500 pA.

**Figure 5:**
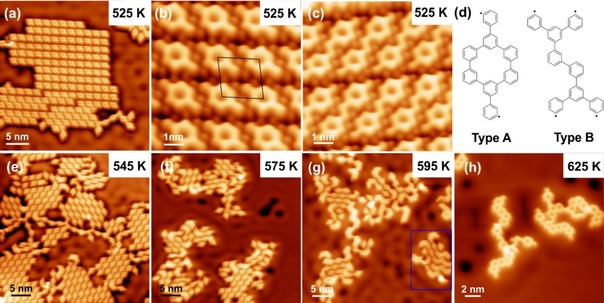
Overview and detailed STM images of covalently‐linked porous *m*TBPB structures on Ag(111) after deposition at RT and subsequent annealing. (a–c) STM images after annealing to 525 K with self‐assembled *m*TBPB dimers (b) and *m*TBPB tetramers (c). (d) Possible structures for covalently‐linked *m*TBPB dimers, which feature a closed pore (Type A) and no pore (Type B). Type B dimers were only observed a handful of times. Formation of porous nanoribbons on Ag(111) upon subsequent annealing at (e) 545 K, (f) 575 K, (g) 595 K, and (h) 625 K: (e) Self‐assembled covalently‐linked short polymer chains with periodic pores. (f) Beginning of the nanoribbon formation. (g) Formation of self‐assembled nanoribbons highlighted by the blue frame. (h) Interconnected nanoribbons after desorption of Br. STM parameters: (a) 150 pA, 100 mV; (b) 300 pA, 50 mV; (c) 156 pA, 9 mV; (e) 200 pA, 100 mV; (f, g) 250 pA, 100 mV; (h) 250 pA, 50 mV.

After annealing to 550 K, the Ag adatoms are released and the C−C coupling is achieved on Ag(111) and Ag(100). The STM images in Figure 4 for Ag(100) and Figure 5 for Ag(111) show the formation of polymer chains composed of CHP rings bridged by two *m*‐phenylene units. The pore‐to‐pore distance of 2.02 nm±0.05 nm along the chains is on both Ag surfaces the same and confirms the completion of the Ullmann‐type coupling. While on Ag(111) the self‐assemblies of the prochiral porous polymer chains are well‐ordered,^7^ we observe a loss of structural quality on Ag(100), see Figure 4a‐b. The interdigitated Br atoms are inhomogeneously distributed on Ag(100), which weakens the interchain interactions *via* H−Br bonding.^14^ This might be related to competing molecule‐surface and intermolecular interactions or a mismatch of preferred adsorption sites for the Br on the Ag(100) surface. Figure 4b shows an example of alternating left‐ and right‐handed polymer chains and confirms that the islands are heterochiral on Ag(100). However, most islands show irregularly distributed left‐ and right‐handed polymer chains. In contrast, the Br−H hydrogen bonding is strong enough on Ag(111) that the prochiral polymer chains assemble in enantiomer‐pure, left‐ and right‐handed, domains.^7^ A similar chiral resolution due to Br−H hydrogen bonding has also been observed for chiral tris‐helicene in Ullmann‐type coupling reactions.^15^ Nonetheless, the self‐assembled chains are composed of *C_s_*‐*trans* dimers on both surfaces that provide the closest packed self‐assembly. The self‐assembled polymers chains are of finite length on both surfaces, which is essential for the formation of the porous nanoribbons upon further annealing. Apart from the self‐assembled porous polymer chains, curved and branched polymer chains, as well as sparse hexagonal aggregates composed of six *m*TBPB, can be found in overview STM images on Ag(100), see Figure S2. The sparse hexagonal aggregates are mostly composed of *C_3h_*‐conformers and have not been observed on Ag(111).

Upon annealing to 630 K on Ag(100), the formation of zigzag‐shaped nanoribbons with a well‐defined width and periodic pores is frequently observed, see Figure 4c. The nanoribbons are randomly ordered and often interconnected in a network. In interlinked chains, the flipping of phenylene units and the closure of CHP rings *via* dehydrogenation reactions is often limited by the strain that occurs through the shortening of the chains in the last reaction step. Therefore, not all polymer chains transform into porous nanoribbons. A zoom image of the zigzag‐shaped nanoribbon structure, see Figure 4d, confirms the formation of the porous nanoribbons on Ag(100) with a uniform pore spacing of 0.8 nm±0.1 nm. Hence, the covalent reaction steps on Ag(100) lead to comparable results as on Ag(111).

### 2.3 Formation of Free‐Standing Nanoribbons on Ag(111) by Kinetic Control

On both Ag surfaces, the maximal length of the nanoribbons is limited due to the interlinking of the porous chains and the subsequent formation of a 2D network. Therefore, we studied the reaction kinetics in detail by looking at the early stage of the covalent chain formation to create free‐standing nanoribbons. Sub‐monolayer amounts of *m*TBPB were deposited on Ag(111) at RT and subsequently annealed to 525 K. In the STM images in Figure 5, we observe self‐assembled islands of short covalently‐linked chains with mostly one, two, three, or a few pores. The chains interact *via* H−Br bonding^14^ with the adsorbed Br atoms, which are released during the Ullmann‐type coupling and adsorb in‐between the chains. The unit cell size for the assembly of one‐pore chains (covalent *m*TBPB dimers) in Figure 5b measures *a*=1.6±0.1 nm, *b*=1.8 nm±0.1 nm and *θ*=96.7°±3°. There are six orientations (three for each enantiomer) of the prochiral chains, which is presumably templated by the three‐fold Ag lattice symmetry.

Two types of covalently‐linked *m*TBPB dimers can potentially be formed, see Figure 5d. Type A dimers have a pore, while type B dimers show no pore. We note that *cis* or *trans* configurations of type A dimers are challenging to identify in these short chains unambiguously. However, the asymmetry of the Br adsorbed at both ends suggests mostly *trans* configurations, in line with longer chains. Surprisingly, the short chains are composed mostly of dimers from type A, which feature pores, see Figure 5. This might be templated by the organometallic intermediates, where the organometallic version of the type A dimers (*C_s_*‐dimers in Figure 3) are commonly observed. The observation of type A dimers is also consistent with the exothermic character of the Ullmann‐type reaction on Ag(111)^16^ as twice the energy is gained upon the C−C coupling for type A dimers with two new C−C bonds formed opposed to one in the type B dimers. Accordingly, the covalent chains in the early reaction stage contain an even number of molecules with closed pores. The substrate likely saturates the radicals at the end of the chains as we do not observe metal adatoms at the end of the chains.

The formation of the porous nanoribbons starts on Ag(111) at around 575 K, as observed at the edges of the self‐assembled islands in Figure 5f. For the formation of the porous nanoribbons, the covalently‐linked porous chains can close the remaining pores by flipping an *m*‐phenylene unit followed by a C−C bond formation through a dehydrogenation reaction, see Figure S3. The yield of the nanoribbon formation is high at these reaction temperatures because the polymer chains feature open ends.^7^ Hence, strain by reducing the polymer length when going from the porous chains to the nanoribbons is avoided. In 2D networks composed of interconnected polymer chains, the strain can limit or bias the flipping. This is observed by annealing directly to higher temperatures, see Figure S4. We note that the short porous nanoribbons at 595 K also form small self‐assembled patches (see blue frame Figure 5g). The Br is mostly desorbed from the Ag(111) at around 625 K in our measurements, as seen in Figure 5h. At this temperature, the nanoribbon formation through dehydrogenation reactions is concluded and the ribbons are interconnected. Hence, the Br desorption proceeds in a similar temperature range as the dehydrogenation reactions, which has previously been reported on Au(111).^17^ In conclusion, the preparation protocol allows to optimize the reaction product as follows: At lower reaction temperatures (575 K–595 K) well‐ordered short nanoribbons of finite size can be synthesized. Instead, annealing directly to higher temperatures (630 K, 10 K/min) leads to longer nanoribbons, which are, however, interconnected in 2D networks as previously reported,^7^ see Figure S4.

## 3 Conclusions

The on‐surface synthesis of porous chains by an Ullmann‐type coupling reaction and the subsequent formation of porous nanoribbons *via* dehydrogenation reactions have been demonstrated for the conformationally flexible *m*TBPB on Ag(100). The templating effect of the lattice symmetry leads to different self‐assembled organometallic intermediates on Ag(100) vs. Ag(111) surfaces. Thereby, the preferred adsorption sites of the metal adatoms act as a template. In contrast, the lattice symmetry has a minor impact on the structure of the covalent reaction products. Also, the reaction temperatures are approximately the same on both Ag surfaces, which indicates comparable reaction barriers. In conclusion, we found: (a) The linear intermolecular C−Ag−C bonding motif in the intermediate organometallic structures facilitates the high selectivity towards *C_s_*‐dimers on both surfaces, which are the building blocks for the porous nanoribbon. (b) The preferred adsorption sites of the metal adatoms drive the self‐assembly of the organometallic intermediate structures. (c) Despite different organometallic structures on Ag(100) and Ag(111), we observe similar covalent porous polymers and porous nanoribbons. Moreover, a detailed analysis of the reaction kinetics of *m*TBPB on Ag(111) unveiled that annealing at moderate reaction temperatures leads to short free‐standing porous nanoribbons of a finite size that self‐assemble in islands. Instead, annealing directly to higher reaction temperatures leads to longer nanoribbons, which are however interconnected in 2D networks.

In conclusion, our findings not only illustrate the role of surface templating effects and conformational changes in the on‐surface synthesis of porous nanoribbons but also showcases that Ullmann‐type reactions successfully proceed on Ag(100).

## Experimental Section

### STM Measurements

The experiments were performed in a two‐chamber ultra‐high vacuum (UHV) system, which operates at a base pressure below 1 ⋅ 10^−10^ mbar. We used a low‐temperature scanning tunneling/atomic force microscope from Scienta‐Omicron GmbH. The STM measurements were carried out in constant‐current mode at a sample temperature of 4.7 K. In the experiment, the bias voltage is applied to the tip, while the sample is grounded. However, the bias voltages mentioned in the text are given with respect to a grounded tip. Mechanically‐cut Pt/Ir tips (90 % Pt, 10 % Ir) prepared by controlled indentation into the metal surface were used for topographic measurements. The STM images were analyzed with the WSxM software.^18^ The lattice directions on Ag(100) were determined by low‐energy electron diffraction (LEED) measurements.

### Sample Preparation

The Ag(111) and Ag(100) (from MaTecK) were cleaned by repeated Ar^+^ ion sputtering and annealing cycles (1 keV, 750 K for Ag(111) and 670 K for Ag(100)). The 1,3,5‐tris(3‐bromophenyl)‐benzene (TCI, purity >96 %), see Scheme 1, was thermally evaporated with a rate of 0.1 ML/min from a commercial Knudsen cell (Kentax GmbH) with the quartz crucible held at 415 K. The molecules were thoroughly degassed prior deposition. The evaporation rate was determined by a quartz crystal microbalance. The metal substrate was kept at room temperature (300 K) during the deposition of the molecules and was subsequently heated to the indicated temperatures to initiate reactions.

## Conflict of interest

The authors declare no conflict of interest.

## Supporting information

As a service to our authors and readers, this journal provides supporting information supplied by the authors. Such materials are peer reviewed and may be re‐organized for online delivery, but are not copy‐edited or typeset. Technical support issues arising from supporting information (other than missing files) should be addressed to the authors.

Supplementary
