# Supplementary material for: On‐Surface Synthesis of Porous Carbon Nanoribbons on Silver: Reaction Kinetics and the Influence of the Surface Structure
Source: Chemphyschem. 2019 Sep 3;20(18):2333–9. doi: 10.1002/cphc.201900347 (PMC6771863; doi:10.1002/cphc.201900347)
Supplement: Supplementary file 1 — Supplementary [file CPHC-20-2333-s001.pdf]

CHEMPHYSCHEM

Supporting Information

© Copyright Wiley-VCH Verlag GmbH & Co. KGaA, 69451 Weinheim, 2019

On-Surface Synthesis of Porous Carbon Nanoribbons on Silver: Reaction Kinetics and the Influence of the Surface Structure

Maximilian Ammon, Martin Haller, Shadi Sorayya, and Sabine Maier*©2019 The Authors. Published by Wiley-VCH Verlag GmbH & Co. KGaA.
This is an open access article under the terms of the Creative Commons Attribution License, which permits use, distribution and reproduction in any medium, provided the original work is properly cited.An invited contribution to a Special Issue on On-Surface Synthesis

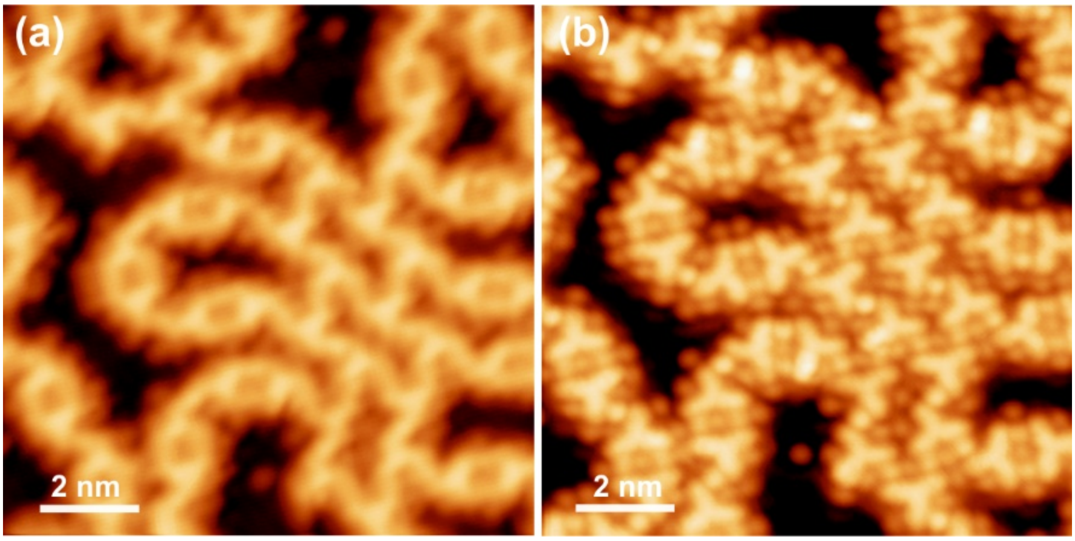

Figure S1. Organometallic structures of the debrominated *m*TBPB on Ag(100). Difference between an unfunctionalized (likely metallic) tip and a functionalized tip (likely Br-terminated). The functionalized tip images the adsorbed Br and Ag adatoms sharper. STM parameters: (a-b) -50 mV and 50 pA.

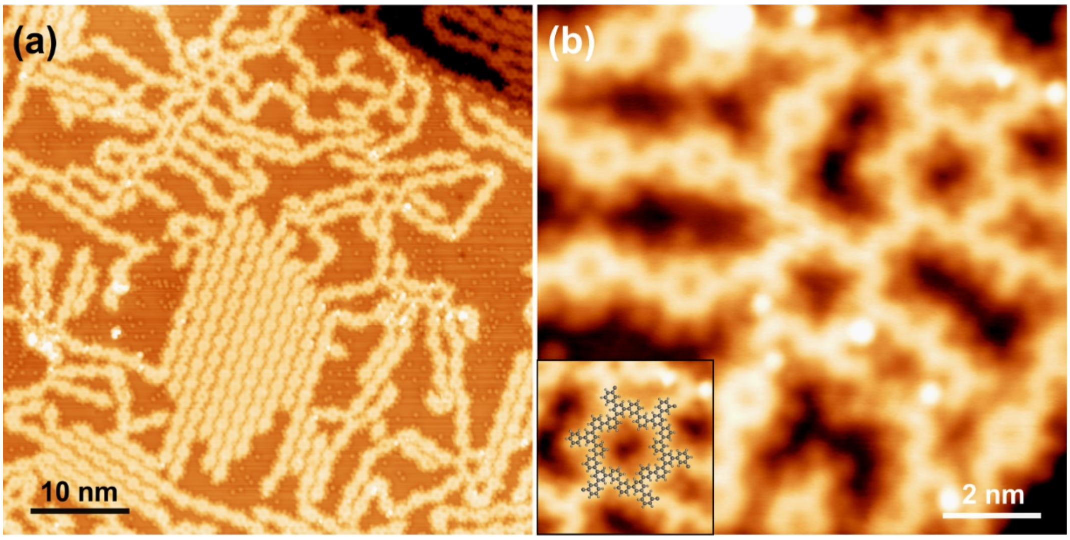

Figure S2. Overview (a) and detailed (b) STM image showing the covalently-linked porous polymer chains on Ag(100) after annealing at 350 K. (b) Sparse hexagonal aggregates composed of six *m*TBPB that are mostly composed of C_{3h} conformers. The inset shows as a guide to the eye the overlaid structure. STM parameters: (a) 2.7 mV, 50 pA, (b) -50 mV, 500 pA.

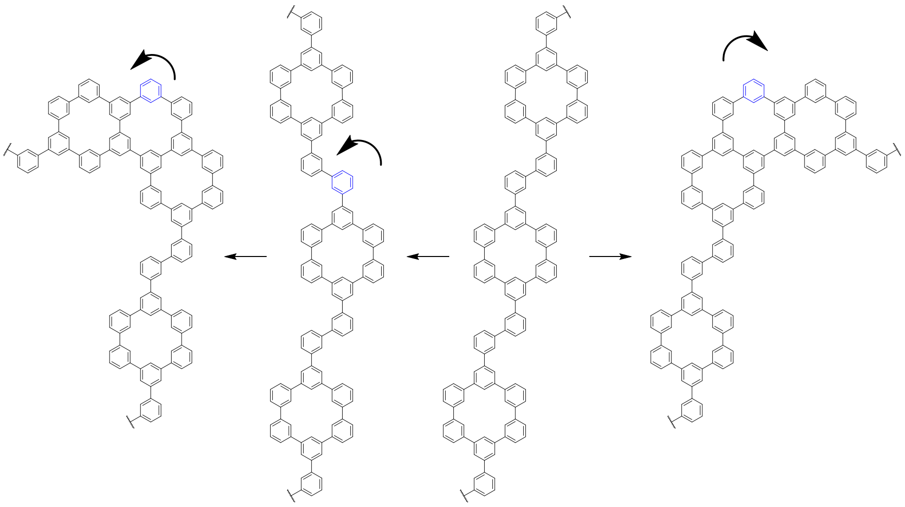

Figure S3. Schematics depicting the initial steps in the reaction pathway of nanoribbons from the porous polymer chains. Closing the additional pores in the same direction as the chirality of the chain requires a single flipping event per additionally closed pore if we assume that the closing of the additional pore is facilitated by the isomerization reaction a single *m*-phenylene unit. The opposite closing direction in comparison to the chain's chirality would require that both m-phenylene units need to flip: the first would change the chirality and the second would close the additional pore.

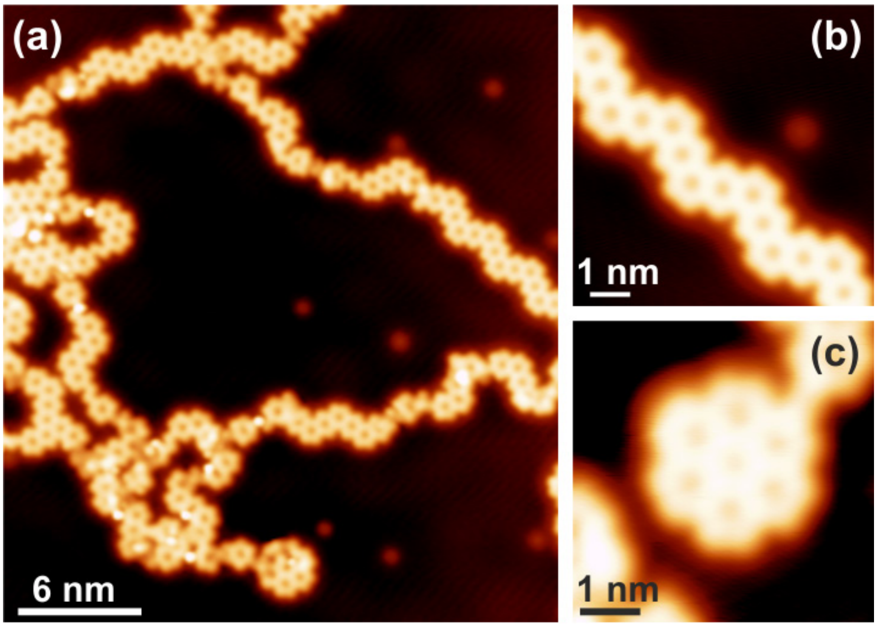

Figure S4. Overview STM image showing the formation of covalently linked porous nanoribbons on Ag(111) after annealing at 630 K. The porous nanoribbons are interconnected in a randomly ordered 2D network (b) Detailed image of a porous nanoribbon. (c) Detailed image of a porous graphene nanodot composed of seven CHP rings after a complete dehydrogenation reaction. STM parameters: (a-b) -520 mV and 160 pA, (c) -1 V, 300 pA.
